# Sex-specific prolactin disturbance and divergent gonadal hormone correlates in first-episode schizophrenia

**DOI:** 10.1093/ijnp/pyaf068

**Published:** 2025-09-13

**Authors:** Anle Pan, Jindong Wang, Jing Liang, Meihong Xiu, Qiang Hu, Shuangli Zhang

**Affiliations:** The Second People's Hospital of Lishui, Zhejiang Provincial Clinical Research Center for Mental Disorders, Zhejiang, China; Qingdao Mental Health Center, China; Qingdao Mental Health Center, China; Peking University HuiLongGuan Clinical Medical School, Beijing Huilongguan Hospital, Beijing, China; Department of Psychiatry, Zhenjiang Mental Health Center, Zhenjiang, Jiangsu Province, China; Department of Psychiatry, Wuhu Hospital of Anding Hospital (The Fourth People's Hospital of Wuhu), Anhui, China; Department of Psychiatry, The Third Hospital of Quzhou, Quzhou, Zhejiang Province, China

**Keywords:** schizophrenia, first episode, gonadal hormones, prolactin

## Abstract

**Background:**

Hyperprolactinemia and altered prolactin (PRL) levels are well-documented in schizophrenia. However, very few studies have investigated sex-specific differences in the prevalence of PRL disturbances in first-episode patients with schizophrenia. This cross-sectional study investigated sex-specific PRL dysregulation and its interplay with gonadal hormones in first-episode schizophrenia (FES) patients.

**Methods:**

One hundred eighty-nine first-episode patients (96 males, 93 females) with minimally treated (≤2 weeks) were recruited. PRL levels and gonadal hormones were measured in all participants.

**Results:**

We found a significantly higher prevalence of abnormal PRL levels in males compared to females (32.3% vs 8.6%, χ^2^ = 16.2, *P* < .001). Comparative analysis of gonadal hormones between elevated PRL (*n* = 39) and normal PRL (*n* = 150) groups demonstrated elevated follicle-stimulating hormone (*Z* = 2.7, *P* = .007) and testosterone (*Z* = 3.7, *P* < .001) in the hyperprolactinemic group. In the elevated PRL group, PRL positively correlated with progesterone and testosterone, whereas in the normal PRL group, PRL showed positive associations with estradiol and luteinizing hormone, but negative correlations with progesterone.

**Conclusions:**

Our findings underscore the complex and sex-specific nature of PRL dysregulation and its association with gonadal hormones in FES patients.

**Significance Statement**

This work analyses the sex-specific pattern of prolactin (PRL) disturbance in antipsychotic-naïve or minimally treated first-episode schizophrenia (FES), the earliest clinical manifestation of the disorder. While hyperprolactinemia is well documented, its sex-stratified links to gonadal hormones at illness onset remain unclear. We therefore reassessed PRL and gonadal hormone levels in 189 drug-naïve or minimally treated FES patients. Results showed that abnormal PRL levels were almost four times more common in males than in females. Hyperprolactinemic patients displayed elevated testosterone and follicle-stimulating hormone, whereas normal-PRL patients exhibited inverse PRL-progesterone relationships. These findings reveal a sex-divergent neuroendocrine signature at psychosis onset, underscoring the need for sex-specific endocrine monitoring and personalized early intervention strategies that target PRL-gonadal hormone pathways.

## INTRODUCTION

Schizophrenia impacts 1% people worldwide, yet its underlying causes remain poorly understood.^1^ Emerging research points to a complex interplay of inherited traits and environmental factors disrupting neuroendocrine pathways, particularly the pituitary-hypothalamus-adrenal axis hormone-related brain pathways,^2^ which may worsen neurodevelopmental changes linked to genetic risks. This dysregulation of stress-related hormonal systems could drive the progressive neural changes observed in schizophrenia.^3^

Increased prolactin (PRL) levels and hyperprolactinemia are well-documented in subjects with schizophrenia,^4-6^ including among first-episode patients before antipsychotic exposure.^7-9^ Specifically, a recent review and meta-analysis reported that drug-naïve individuals with first-episode schizophrenia (FES) exhibit altered blood concentrations of adrenocorticotropic hormone (ACTH), PRL, and thyroid-stimulating hormone (TSH).^10^ While antipsychotic medications-particularly dopamine D_2_ receptor antagonists such as risperidone and haloperidol-remain a well-established contributor to PRL dysregulation resulting from direct blockade of pituitary dopamine receptors,^5^^,^^11^ emerging evidence posits intrinsic disease-related mechanisms as an additional etiological factor. Studies report mild hyperprolactinemia in a subset of antipsychotic-naïve FES patients, potentially linked to stress-induced hypothalamic–pituitary–adrenal (HPA) axis hyperactivity or genetic variants affecting PRL regulation pathways.^12^^,^^13^ These findings highlight the need to disentangle medication effects from neuroendocrine alterations inherent to schizophrenia pathology.

Relatively few studies have been conducted on gonadal hormones in patients with schizophrenia.^14^^,^^15^ Emerging evidence highlights sex-specific endocrine disturbances in schizophrenia, with gender-dimorphic hormonal profiles linked to symptom expression.^16–18^ Premenopausal females with FES exhibit significantly lower serum estrogen (E2) levels compared to healthy controls, and physiological E2 fluctuations (eg, during menstrual luteal phases or postpartum periods) correlate with psychotic symptom exacerbations,^19–21^ potentially mediated by estrogen’s modulation of dopaminergic and glutamatergic pathways. In males, a bimodal testosterone (T) distribution is observed: hypoprolactinemia (low T) inversely associates with negative symptom severity, while hyperandrogenism (elevated T) correlates with heightened positive symptoms.^16^^,^^22^^,^^23^

Prolactin plays a pivotal role in modulating gonadal hormones.^24^^,^^25^ Hyperprolactinaemia is a well-established cause of infertility in both male and female individuals.^26^ In females, elevated PRL suppresses estrogen synthesis, leading to menstrual irregularities and anovulation.^27^ Mechanistically, increased PRL may disrupt gonadotropin-releasing hormone (GnRH) neurons in the hypothalamus or pituitary gland,^28^ thereby affecting the secretion of gonadotropins, luteinizing hormone (LH), and follicle-stimulating hormone (FSH). Notably, infertility associated with hyperprolactinaemia can be reversed through pulsatile GnRH replacement therapy.^29^ Further, hyperprolactinemia blocks castration-induced upregulation of GnRH mRNA expression in rats,^30^ underscoring its central role in disrupting hypothalamic–pituitary–gonadal (HPG) axis regulation.

Building upon evidence that hyperprolactinemia disrupts GnRH-regulated hormones, this study aimed to investigate whether elevated PRL levels are associated with aberrant gonadotropin (LH/FSH) and sex steroid (E2, progesterone, T) profiles in FES patients with drug-naïve or minimally treated (≤2 weeks). By recruiting a cohort of FES individuals for baseline endocrine evaluations, we sought to: (1) establish disease-specific correlations between PRL and HPG axis hormones, independent of antipsychotic drug effects; (2) identify potential sex-stratified endocrine disturbances linked to PRL dysregulation; and (3) provide foundational data for future mechanistic studies exploring PRL-GnRH interactions in schizophrenia pathophysiology.

## METHODS

### Subjects

Individuals with FES were consecutively recruited from three public hospitals in China. Participants were either antipsychotic-naïve or had received antipsychotic treatment for fewer than 2 weeks.

Participants were eligible if they were aged 16-45 years, had a DSM-5 schizophrenia diagnosis confirmed via Structured Clinical Interview for DSM-5 (SCID), exhibited normal blood pressure, ECG findings and routine blood tests, including complete blood count (CBC), liver function tests (LFTs), renal function tests (RFTs), and fasting glucose levels at screening, and could provide written informed consent. Exclusion criteria included comorbid central nervous system disorders, other DSM-5 psychiatric diagnoses, substance use disorders, intellectual disability, pregnancy/breastfeeding, or the use of oral contraceptives.

The Ethics Committee of Beijing Huilongguan Hospital approved this study, and written informed consent was obtained from each patient.

### Measurement of Gonadal Hormones and PRL

Fasting venous blood samples were collected at 07:30 AM from all participants after a 12-hour overnight fast. Serum was isolated via centrifugation immediately after collection. The biochemical analysis was carried out in duplicate in the hospital laboratory by three technicians blind to the clinical status of the participants. The laboratory adheres to strict quality control measures to ensure the accuracy and reproducibility of results. Gonadal hormones, including E2, FSH, P, LH, and T, as well as PRL, were quantified using chemiluminescence immunoassays on a Beckman UniCel DxI800A analyzer (Beckman Coulter). Quality control samples were included to ensure the accuracy and precision of the measurements. The intra-assay and inter-assay coefficients of variation were maintained below 5% and 10%, respectively, to ensure the reliability of the results.

In this study, reference ranges for PRL were 56-278 mIU/L (male) and 72-566 mIU/L (female). Gonadal hormone reference values were adopted from prior published standards.^15^ The reference values for the male healthy population were as follows: E2 (55.07-142.99 pmol/L), FSH (1.27-19.26 mIU/mL), P (0.45-6.55 nmol/L), LH (1.24-8.62 mIU/mL), and T (6.07-27.1 nmol/L). The reference values for the female healthy population were as follows: E2 (follicular phase: 55.65-543.79 pmol/L; mid-luteal phase:111.38-1006.74 pmol/L; ovulation phase:108-1624.86 pmol/L), FSH (follicular phase: 3.85-8.78 mIU/mL; mid-luteal phase:1.79-5.12 mIU/mL; ovulation phase:4.54-22.51 mIU/mL), P (follicular phase: 0.98-4.83 nmol/L; mid-luteal phase:16.41-59.02 nmol/L), LH (follicular phase: 2.12-10.89 mIU/mL; mid-luteal phase:1.2-12.86 mIU/mL; ovulation phase:19.18-103.03 mIU/mL), and T (0.35-2.60 nmol/L).

### Data Analysis

Data were analyzed using SPSS (version 22.0), with a statistical significance threshold of *α* = 0.05.

Categorical variables are reported as absolute and relative frequencies (%) and analyzed via the *Chi*-square test. The Shapiro–Wilk test and Q-Q plot were used to test normality for continuous variables. Normally distributed continuous variables are reported as mean ± SD and compared using the ANOVA. The continuous variables of non-normal distribution were described using the median and interquartile ranges (IQRs). The non-parametric Mann–Whitney *U* test was performed to compare data between groups.

Spearman’s rank correlation analysis was conducted to evaluate the associations of PRL with gonadal hormones in patients, considering the non-normal distributions of PRL and hormones. Bonferroni correction was used to avoid inflated Type I error for multiple comparisons.

## RESULTS

### Demographic and Clinical Data

A total of 230 patients were screened for eligibility. Thirty patients were excluded for not meeting the inclusion/exclusion criteria, four declined to provide written informed consent, and seven refused to participate. Finally, the study cohort comprised 189 patients with schizophrenia aged 16-45 years (96 males and 93 females). The cohort had a mean duration of illness of 28.7 months (less than 5 years), a mean age at onset of 24.0 years, and a mean BMI of 22.7.

Baseline characteristics such as age of onset, body mass index (BMI), and educational attainment showed no sex-based differences (all *P* > .05). However, female participants were significantly older (*P* = .04) than male participants (Table 1). Additionally, smoking prevalence was markedly higher in male participants (*P* < .001).

**Table 1:** The demographic, gonadal hormones, and prolactin in patients with schizophrenia.

**Characteristics**	**Males** ***n* = 96**	**Females** ***n* = 93**	***Z, F, X*** ^***2***^ **(*P*)**
**Age (ys)**	25.3 ± 7.3	27.6 ± 8.5	4.2(.04)
**Onset age (ys)**	23.1 ± 7.4	25.0 ± 8.5	2.9(.09)
**Education levels (ys)**	12.0 ± 3.3	11.6 ± 3.8	0.6(.45)
**Smokers (*n*, %)**			25.7(<.001)
** Yes (*n*, %)**	30(31.3%)	3(3.2%)	
** No (*n*, %)**	66(68.7%)	90(96.8%)	
**Married (*n*, %)**			13.7(<.001)
** Yes (*n*, %)**	20(20.8%)	43(46.2%)	
** No (*n*, %)**	76(79.2%)	50(53.8%)	
**BMI (kg/m^2^)**	23.1 ± 3.4	22.3 ± 3.4	2.9(.09)
**WC (feet)**	2.5 ± 0.3	2.2 ± 0.2	41.3(<.001)
**PRL (mIU/L)**	241.2 ± 166.2	296.6 ± 204.2	4.2(.04)
**E2 (pmol/L)**	103.00(78.00124.00)	252.50(150.00482.75)	8.7(<.001)
**FSH (mU/L)**	3.60(2.60,5.30)	5.89 ± 2.57	5.2(<.001)
**P (nmol/L)**	1.81(1.14,2.40)	2.75(1.56,15.67)	4.9(<.001)
**LH (mIUl/mL)**	3.60(2.55,4.82)	5.56(3.28,9.55)	4.6(<.001)
**T (nmol/L)**	13.79 ± 4.62	1.88 ± 0.78	11.9(<.001)

Abbreviations: BMI, body mass index; E2, estradiol; FSH, follicular-stimulating hormone; LH, luteinizing hormone; ms months; P, progesterone; PRL, prolactin; T, testosterone; WC, waist circumference; ys, years.

There were 39 patients with abnormal PRL levels (39/189, 20.6%) and 150 patients with normal PRL levels. We also compared the demographic and clinical characteristics between the two groups and found that there were no significant differences in BMI, age, onset age, disease duration, educational levels, and smoking status (all *P* > .05).

### PRL Abnormalities by Sex

The frequencies of abnormal PRL were *n* = 31 (32.3%) in male participants and *n* = 8 (8.6%) in female participants. The difference in the frequencies between males and females was significant (*X*^2^ = 16.2, *P* < .001). PRL levels were lower in male patients than female patients (Z = 2.3, *P* = .02).

### Gonadal Hormone Profiles by PRL Status

In a comparative assessment of the gonadal hormones between patients with elevated PRL levels (*n* = 39) and those with normal PRL levels (*n* = 150), statistically significant differences in FSH levels (Z = 2.7, *P* = .007) and T levels (Z = 3.7, *P* < .001) were observed. Patients in the elevated PRL group had higher levels of FSH and T than those in the normal PRL group (Table 2).

**Table 2:** The gonadal hormones between schizophrenia patients with normal prolactin levels and those with abnormal levels.

**Characteristics**	**Abnormal PRL group (*n* = 39)**	**Normal PRL group (*n* = 150)**	***Z* (*P*)**
**E2 (pmol/L)**	118.0(82.0, 175.0)	142.5(89.5, 263.0)	1.5(.13)
**FSH (mU/L)**	3.5(2.5, 4.8)	5.1(3.2, 6.8)	2.7(.007)
**P (nmol/L)**	1.8(1.2, 3.8)	2.1(1.3, 4.0)	0.9(.33)
**LH (mIUl/mL)**	4.1(3.1, 5.8)	4.1(2.6, 7.3)	0.9(.39)
**T (nmol/L)**	12.9(7.7, 15.6)	2.8(1.6, 12.0)	3.7(<.001)

Abbreviations: E2, estradiol; FSH, follicular-stimulating hormone; LH, luteinizing hormone; P, progesterone; T, testosterone.

### PLR-Gonadal Hormone Correlations

In patients with elevated PRL levels, positive associations were observed between PRL levels and P (*r* = 0.38, *P* = .02) or T (*r* = 0.44, *P* = .005) (Table 3). After Bonferroni correction, the association between PRL and T remained significant. Among individuals with normal PRL levels, there were significant positive associations between PRL levels and E2 (*r* = 0.33, *P* < .001) and LH (*r* = 0.32, *P* < .001) and negative associations between PRL and P (*r* = -0.25, *P* = .002). After Bonferroni correction, all these associations between PRL and E2, LH, and P remained significant.

**Table 3:** Associations between prolactin levels and gonadal hormones in patients with schizophrenia.

	**Prolactin levels**			
	**Abnormal group**		**Normal group**	
	*r*	*P*	*r*	*P*
**E2 (pmol/L)**	0.26	.11	0.33	<.001
**FSH (mU/L)**	0.06	.71	0.18	.03
**P (nmol/L)**	0.38	.02	0.10	.22
**LH (mIUl/mL)**	0.04	.81	0.32	<.001
**T (nmol/L)**	0.44	.005	−0.25	.002

Abbreviations: E2, estradiol; FSH, follicular-stimulating hormone; LH, luteinizing hormone; P, progesterone; T, testosterone.

## DISCUSSION

This study demonstrated that (1) the rate of male patients with PRL abnormalities was significantly higher than that of females, but female patients had higher overall PRL levels; (2) FSH and testosterone levels were significantly higher in the group with elevated PRL compared to the group with normal PRL; and (3) in the elevated PRL group, PRL correlated with progesterone and testosterone. In the normal PRL group, PRL positively correlated with E2 and LH and negatively correlated with progesterone.

This study revealed that the prevalence of PRL abnormalities was higher in males than in females at the onset of S.Z. Most previous studies have focused on sex-based comparisons of PRL levels,^31^^,^^32^ and few studies have investigated sex-specific prevalence of PRL dysregulation in early psychosis. For instance, the European First Episode Schizophrenia (EUFEST) study reported that higher PRL rates of 71% were identified in early psychosis of schizophrenia, with half of the cases occurring in drug-naïve patients.^33^ Similarly, studies of drug-naïve FES cohorts identified elevated PRL in 29%-39% of patients,^34^^,^^35^ with both sexes showing significant PRL elevations.^7^ However, these studies did not directly compare sex-specific prevalence of PRL abnormalities in patients, limiting insights into gender disparities in endocrine dysfunction at illness onset. Our findings were not consistent with previous studies that found no sex difference in PRL abnormality rates.^35^^,^^36^ This discrepancy may be due to the following variables: differences in sample characteristics, PRL measurements, and stress assessments. Suggesting that the illness itself or stress may influence PRL levels.

In our study, we found that levels of FSH and testosterone were significantly higher in the group with elevated PRL compared to the group with normal PRL. This finding may be related to the regulatory effects of PRL on the HPG axis.^37^ Elevated PRL levels may inhibit the secretion of GnRH from the hypothalamus, subsequently affecting the secretion of FSH, LH, and T. Additionally, stress, which is common in patients with schizophrenia, may directly or indirectly influence the secretion of PRL and sex hormones.^38–41^ In clinical studies, PRL was positively correlated with stress biomarkers (eg, cortisol) in first-episode patients.^42^

Further analysis revealed that in the group with elevated PRL, PRL was positively correlated with P and T. In contrast, in the group with normal PRL, PRL was positively correlated with E2 and LH and negatively correlated with P. These differences likely reflect the distinct regulatory mechanisms of PRL on sex hormones at varying levels. When PRL is elevated, PRL may promote the secretion of P and T through direct or indirect pathways, possibly involving interactions with the HPA axis. In comparison, the positive correlations between PRL and estradiol and LH in the normal PRL group likely reflect its physiological regulatory role in the menstrual cycle. Additionally, our findings of the negative correlation between PRL and progesterone suggest a complex role for PRL in regulating luteal function.^43^ These differential correlations are significant for understanding the endocrine disturbances associated with PRL in patients with schizophrenia and may provide new targets for future therapeutic strategies.

Several limitations should be noted. First, physical activity, diet, and psychosocial stress, known modulators of PRL and gonadal hormone levels, were not systematically measured or adjusted for. This may obscure the true association between PRL and hormonal profiles, particularly given the potential influence of stress-induced PRL elevation in first-episode psychosis. Second, in female participants, PRL and sex hormone levels were not stratified by menstrual cycle phase, introducing variability that may attenuate or bias observed correlations, especially in the normal PRL group. Third, the inclusion of patients with up to 2 weeks of antipsychotic exposure introduces potential confounding, as even short-term antipsychotic use may alter PRL and sex hormone levels. A stringent drug-naïve criterion would enhance internal validity.

In conclusion, our study provides novel insights into the interplay between PRL and gonadal hormones in antipsychotic-naïve or minimally treated FES patients. However, our findings were limited by unmeasured confounders (eg, stress, lifestyle factors) and its cross-sectional design, which restricts causal interpretations. Future longitudinal studies integrating neuroimaging, genetic profiling, and dynamic stress assessments are warranted to unravel the neuroendocrine mechanisms underlying these findings and inform personalized therapeutic strategies.

## Data Availability

The datasets generated and analyzed during the current study are available from the corresponding author upon reasonable request.
